# A phase II study of capecitabine and docetaxel combination chemotherapy in patients with advanced gastric cancer

**DOI:** 10.1038/sj.bjc.6601724

**Published:** 2004-03-23

**Authors:** Y H Park, B-Y Ryoo, S-J Choi, H-T Kim

**Affiliations:** 1 Division of Haematology and Oncology, Department of Internal Medicine, Korea Institute of Radiological and Medical Science, Seoul, Korea

**Keywords:** capecitabine, docetaxel, advanced gastric cancer

## Abstract

Capecitabine and docetaxel have considerable single-agent activity in gastric cancer with distinct mechanisms of action and no overlap of key toxicities. A synergistic interaction between these two drugs is mediated by taxane-induced upregulation of thymidine phosphorylase. We investigated the activity and the feasibility of capecitabine and docetaxel combination chemotherapy in patients with previously untreated advanced gastric cancer (AGC). From September 2001 to March 2003, 42 patients with AGC received 21-day cycles of oral capecitabine (1250 mg m^−2^ twice daily on days 1–14) and docetaxel (75 mg m^−2^ i.v. on day 1). The patients received a total of 164 cycles of chemotherapy. The median age was 53.5 years (range 33–73 years). The overall response rate in the 38 efficacy-evaluable patients was 60% (95% confidence interval, 45–74%). The median progression-free survival was 5.2 months (range, 1.0–15.5+ months) and the median overall survival was 10.5 months (range, 2.9–23.7+ months). The most common grade 3/4 adverse events were hand–foot syndrome (HFS: G3 50%), neutropenia (15%) and leucopenia (12%). Further studies of this combination are clearly warranted, albeit with lower doses of both agents (1000 mg m^−2^ twice daily and 60 mg m^−2^) to reduce the rate of HFS and onycholysis.

Gastric cancer remains one of the most common malignancies worldwide (Parkin *et al*, 1999), and the leading cause of cancer death in Korea (National Statistical Office, 2001). Despite improvements in early diagnosis, many patients diagnosed with gastric cancer are inoperable at the time of initial diagnosis. Advanced gastric carcinoma (AGC) remains an incurable disease with a median survival of only 6–9 months in patients receiving chemotherapy. Therefore, there is a need for more effective systemic therapy to improve the management of patients with AGC.

The semisynthetic taxane docetaxel (Taxotere®) is associated with a high overall response rate, prolonged time to progression (TTP) and acceptable tolerability in AGC, both as monotherapy and in combination (Ridwelski *et al*, 2001; Haller and Misset, 2002). Results from several studies in Europe, the USA and Japan have assessed first-line docetaxel monotherapy in AGC and indicate that overall response rates range from 18 to 24% (Einzig *et al*, 1996; Mai *et al*, 1998; Taguchi *et al*, 1998; Bang *et al*, 2002). A number of studies have also investigated various docetaxel/5-FU combinations in patients with recurrent AGC, with response rates ranging from 28% (bolus 5-FU) to 44% (continuous infusional 5-FU), a median TTP of 5.9 months (bolus), and a median overall survival of 7.7 months (bolus) (Constenla *et al*, 2002; Thuss-Patience *et al*, 2003). Recently, presented data show that docetaxel provides a small but significant survival benefit when added to 5-FU/cisplatin in AGC (Van Cutsem *et al*, 2003). However, poor tolerability and a high rate of toxic deaths in this study make the impact of this triplet combination questionable.

The oral fluoropyrimidine capecitabine (Xeloda®) was designed to generate 5-FU preferentially in tumour tissue and to mimic continuous infusional 5-FU. This tumour selectivity is achieved through exploitation of the significantly higher activity of thymidine phosphorylase in many tumour tissues compared with healthy tissue (Miwa *et al*, 1998; Schüller *et al*, 2000). Capecitabine 1250 mg m^−2^ twice daily on days 1–14 every 3 weeks has been shown to be active (overall response rate 28%; stable disease 36%) and well tolerated in a recent phase II study of previously untreated patients with AGC (Hong *et al*, 2002). A 4-weekly intermittent schedule of capecitabine (828 mg m^−2^ twice daily for 3 weeks followed by 1 week of rest) has also been shown to produce overall response rates of 24% in a small, Japanese pilot phase II study in patients with AGC (Koizumi *et al*, 2003). In a larger Japanese clinical trial of 60 patients with previously untreated AGC, the same intermittent schedule led to a response rate of 26% and a median survival of 8.8 months (Kondo *et al*, 2003).

Preclinical studies in human cancer xenograft models demonstrated that administration of docetaxel or paclitaxel results in further upregulation of thymidine phosphorylase in human tissue (Sawada *et al*, 1998). Coadministration of capecitabine or taxanes in xenograft models resulted in synergistic antitumour activity, whereas taxanes in combination with either 5-FU or uracil plus tegafur demonstrated only additive efficacy (Sawada *et al*, 1998). Since capecitabine mimics continuous infusional 5-FU and its safety profile differs from that of docetaxel with little overlap of key toxicities, capecitabine combined with docetaxel is a more compelling and convenient alternative to 5-FU/docetaxel.

The combination of docetaxel with other drugs, especially 5-FU and cisplatin, is pharmacologically feasible in the management of AGC and a number of regimens have been investigated in clinical trials (Haller and Misset, 2002). Early data have also shown high efficacy and manageable safety of 5-FU or capecitabine in combination with docetaxel and cisplatin as first-line therapy (Kang *et al*, 2003; Van Cutsem *et al*, 2003), which indicates the feasibility of triple combinations in this setting. The potential to improve safety and convenience by removing cisplatin to leave a doublet (e.g. capecitabine and docetaxel) is also of interest in AGC, particularly in light of the proven survival benefit of adding capecitabine to docetaxel in second-line metastatic breast cancer (O'Shaughnessy *et al*, 2002). Therefore, the objective of the current phase II study was to investigate the feasibility, efficacy and safety of capecitabine plus docetaxel in patients with previously untreated AGC. We used the dosing schedule as described in a previous phase I study for patients with advanced solid tumours (Pronk *et al*, 2000).

## MATERIALS AND METHODS

### Patients

Patients were eligible if they had histologically confirmed advanced or metastatic gastric adenocarcinoma with bidimensionally measurable disease. This was defined as at least one tumour lesion measuring ⩾1.5 × 1.5 cm with clearly defined margins on spiral CT scan, MRI or abdominal ultrasound. Patients were ⩾18 years of age with an Eastern Cooperative Oncology Group (ECOG) performance status of 0–2, and had received no prior chemotherapy for metastatic disease. Adequate haematological (absolute neutrophil count >1500 *μ*l^−1^, platelets >100 000 *μ*l^−1^), hepatic (total bilirubin <1.5 mg dl^−1^, transaminase levels <3 times the upper normal limit (UNL) or <5 times the UNL in cases of hepatic metastases) and renal (creatinine <1.5 mg dl^−1^) functions were required. The protocol was approved by the institutional review board of the Korean Institute of Radiological and Medical Science, and all patients gave written informed consent before enrolment.

### Treatment schedule

Docetaxel 75 mg m^−2^ was administered as a 1-h intravenous infusion on the first day of each 3-week cycle following premedication with dexamethasone 8 mg twice daily on days 0–2 for 3 days. Capecitabine was administered orally at a dose of 1250 mg m^−2^ twice daily according to the standard intermittent schedule (14 days of treatment followed by a 7-day rest period). Pyridoxine 300 mg day^−1^ was administered orally for 14 days for the prevention of hand–foot syndrome (HFS).

Patients received at least two courses of capecitabine/docetaxel unless rapid disease progression occurred after the first or second course. Patients with response or stable disease received treatment up to a maximum of six cycles or until progressive disease occurred.

### Dose modification for adverse events

Capecitabine treatment interruption or dose reduction was not indicated for reactions unlikely to become serious or life threatening, or for grade 1 toxicity (NCI-CTC, version 2.0). Treatment was interrupted in cases of grade 2 or higher events (with the exception of alopecia, nausea or vomiting and anaemia) and was not resumed until the adverse effect resolved or improved to grade 1 or 0. Capecitabine dose reduction was not required at the first occurrence of a grade 2 event. Capecitabine dose was reduced by 25% for patients who experienced a second occurrence of a given grade 2 event or any grade 3 event. Capecitabine doses were reduced by 50% for patients who experienced a third occurrence of a given grade 2 event, a second occurrence of a given grade 3 event, or any grade 4 event. Treatment was discontinued if, despite dose reduction, a given adverse event occurred for a fourth time at grade 2, a third time at grade 3, or a second time at grade 4. If an adverse event did not improve to grade 1 or less after 3 weeks, the affected patient was withdrawn from the study.

Docetaxel treatment was interrupted in cases of grade 2 or higher event and was not resumed until the adverse effect resolved or improved to grade 1 or 0. For patients who had developed grade 4 neutropenia for >7 days, or was associated with a temperature of >38°C, a 25% permanent dose reduction was required. Patients with grade 4 thrombocytopenia were retreated with a 25% dose reduction after recovery. Treatment was discontinued in cases of grade 3/4 neuropathy. Patients who developed hepatic function abnormalities during therapy received a 25% dose reduction if there was an increase in AST, ALT or alkaline phosphatase of between 2.5 and 5 × UNL. Elevations of any of these enzymes to more than 5 × UNL required docetaxel treatment to be suspended for a maximum of 3 weeks; the patient was taken off the study if they did not recover within that time frame.

### Evaluation criteria

A physical examination, including a neurological examination and complete blood counts, was performed before the first treatment cycle. Pretreatment evaluation also included biochemical analyses, chest X-ray and CT scans to define the extent of the disease. Complete blood cell counts with differential and serum biochemistry analyses were repeated at each treatment cycle. Response was assessed radiologically every two cycles or when progression was suspected. Evaluations were performed by physical examination, chest X-ray, abdomen-pelvis CT scan or ultrasonography. Complete response (CR), partial response (PR), stable disease (SD) and progressive disease (PD) were defined according to WHO criteria.

### Statistical analysis

The trial was designed using Gehan's two-stage testing procedure. Assuming a true response rate of ⩾10%, 22 patients were initially included. If at least one response was observed, enrolment would then continue to 30 evaluable patients, with a target minimum response of 30% and a maximum width of 36% for the 95% confidence interval (CI). However, the number of patients enrolled was increased to 42 to provide a more accurate estimate of response rate. Progression-free survival (PFS) was calculated from the first day of chemotherapy until the date of progression. Overall survival was calculated from the start of the study treatment until death. Progression-free survival and overall survival curves were generated using the Kaplan–Meier method. Response duration was calculated from the date of response confirmation to the date of disease progression.

## RESULTS

### Patient characteristics

A total of 42 patients were enrolled between September 2001 and March 2003. Baseline patient characteristics are listed in Table 1

**Table 1:** Patient characteristics

**Characteristic**	**Number of patients**	**%**
*Number of patients enrolled*	42	
Evaluable for response	38	
Evaluable for safety	41	
Insufficient follow-up data	4	
*Age (years)*		
Median	53.5	
Range	33–73	
*Sex*		
Male	23	55
Female	19	45
*ECOG performance status*		
0	2	5
1	35	83
2	5	12
*Metastatic site*		
Liver	15	36
Lung	3	7
Abdominal lymph node	28	67
Neck node	10	24
Peritoneum	5	12
Bone	3	7
*Number of metastatic sites*		
1	15	36
2	20	48
⩾3	7	17

. Of these, 41 patients were evaluable for safety and 38 for tumour response. The median age of the 19 female and 23 male patients was 53.5 years (range 33–73 years), and most of the patients (88%) had a good performance status (ECOG 0 or 1). In all, 27 patients (64%) had multiple metastases involving two or more organ systems. The most common metastatic site was the abdominal lymph nodes (67%), and the most common metastatic organ was the liver (36%). The median duration of follow-up at the time of this analysis was 15.2 months (range 8.7–35.1 months).

### Response to treatment

A total of 38 patients were evaluable for response. The remaining four patients were not evaluable because of insufficient follow-up data. The overall response rate was 60% (95% CI, 45–74%, Table 2

**Table 2:** Response to treatment

**Response**	**Number (*n*=42)**	**%**
Confirmed response	23	60
CR	2	5
PR	21	55
SD	6	14
PD	7	17
Not assessable	4	10

**Figure 1:**
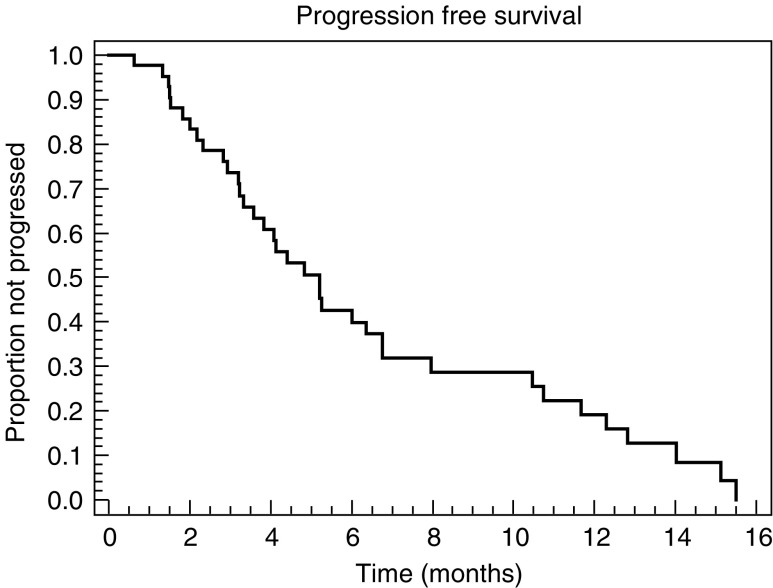
Progression-free survival (*n*=42).

**Figure 2:**
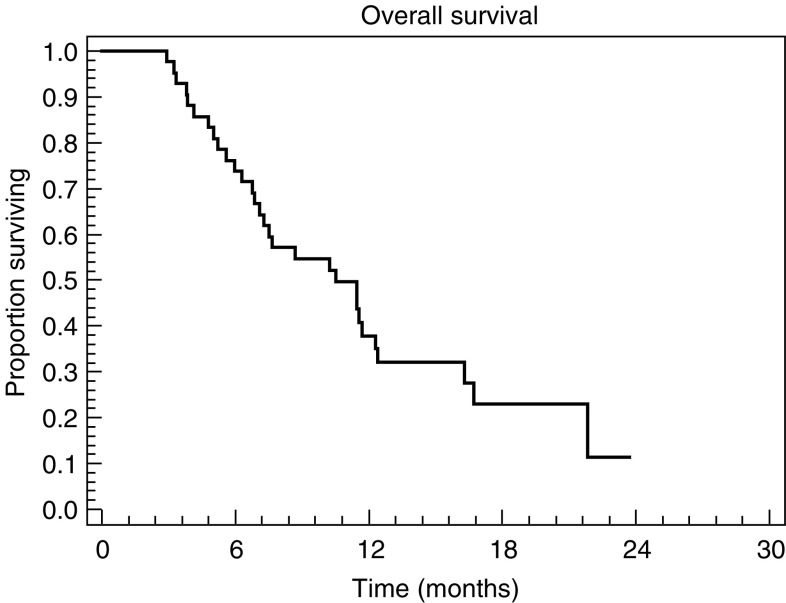
Overall survival (*n*=42).

CR=complete response; PR=partial response; SD=stable disease and PD= progressive disease.

). Two (5%) patients achieved CR confirmed by gastroscopic biopsy. A total of 21 (55%) PRs were observed. The median duration of response in the 23 responding patients was 6.8 months (range 2.2–15.5 months). Six patients (14%) had disease stabilisation, and seven (17%) progressed while on treatment. The median PFS and overall survival were 5.2 months (range 1.0–15.5+ months) (Figure 1

) and 10.5 months (range 2.9–23.7+ months) (Figure 2

), respectively, and the 1-year survival rate was 31%.

### Safety

A total of 164 treatment cycles (median 4, range 1–6 cycles) were administered, of which one was administered to the one patient who was lost to follow-up. Haematological and nonhaematological adverse events associated with treatment are listed in Table 3

**Table 3:** Haematological adverse events

	**Grade (% of patients)**			
	**1**	**2**	**3**	**4**
Anaemia	40	22	7	0
Leukopenia	29	36	10	2
Neutropenia	36	24	5	10
Thrombocytopenia	10	12	7	0

and Figure 3

**Figure 3:**
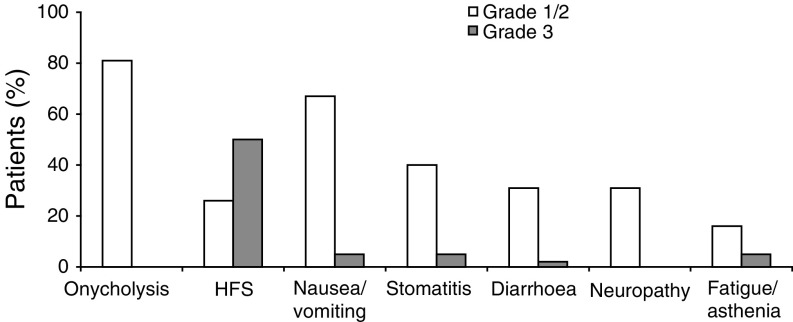
Most common treatment-related adverse events (>20% of patients).

, respectively. The most common clinical adverse events (all grades) were onycholysis (81%), HFS (76%), nausea/vomiting (72%), stomatitis (45%) and neuropathy (31%). However, with the exception of grade 3 HFS, which was seen in 50% of patients, grade 3/4 clinical adverse events were rare. While grade 1/2 haematological adverse events were relatively common, grade 4 neutropenia was reported in only four patients (10%), neutropenic fever in only three patients (7%) and grade 3 anaemia or thrombocytopenia were observed in 7% of patients each. There were no treatment-related deaths.

Treatment delays or dose reductions were necessary in 57 out of 164 cycles. Doses were reduced in 52 cycles (32%) as a result of neutropenia (9%), HFS (22%) and asthenia (1%). In addition, 26 patients (62%) had dose reductions for HFS. Treatment was discontinued in two patients because of three episodes of grade 3 HFS. Treatment was delayed in five cycles (3%). The median dose intensity for capecitabine and docetaxel were 84 and 88%, respectively.

## DISCUSSION

Previous large phase III studies comparing capecitabine with bolus 5-FU plus leucovorin as first-line therapy for metastatic colorectal cancer have demonstrated the high single-agent activity and favourable safety profile of capecitabine over 5-FU in a common gastrointestinal cancer (Hoff *et al*, 2001; Van Cutsem *et al*, 2001; Cassidy *et al*, 2002; Twelves *et al*, 2002). In addition, capecitabine-based therapy has been shown to be active in first- and second-line AGC, achieving response rates in the range of 20–55% (Kim *et al*, 2002; Hong *et al*, 2002; Tebbutt *et al*, 2002; Kang *et al*, 2003; Koizumi *et al*, 2003). The addition of capecitabine to docetaxel has also been shown to produce significant improvements in response rate, TTP and overall survival with a manageable safety profile in metastatic breast cancer (O'Shaughnessy *et al*, 2002; McDonald and Miles, 2003).

In our study, we found that the combination of docetaxel plus capecitabine is highly active as first-line chemotherapy for AGC. The response rate (60%), PFS (5.2 months) and overall survival (10.5 months) in this trial were at the higher end of the range reported in other phase II studies of doublets in AGC (Jeen *et al*, 2001; Cho *et al*, 2002). As would be expected, our results compare favourably with findings on various docetaxel/5-FU combinations in patients with recurrent AGC, where response rates ranged from 28% (bolus 5-FU) to 44% (continuous infusional 5-FU) (Constenla *et al*, 2002; Thuss-Patience *et al*, 2003). In these studies of pretreated patients, median TTP was approximately 5.9 months (bolus), and median overall survival was around 7.7 months (bolus). While the response rate achieved with docetaxel/capecitabine in our study was good, the PFS and overall survival times might have been expected to be higher. There were two potential reasons for this: firstly, the maximum treatment duration was limited to six cycles; secondly, the proportion of patients with HFS (all grades) was very high (76%; grade 3, 50%), despite administration of 300 mg pyridoxine. This resulted in a reduction in dose intensity of both agents. Therefore, we would recommend starting dosing with lower doses of each drug (e.g. capecitabine 1000 mg m^−2^ twice daily; docetaxel 60 mg m^−2^), which have been proven to be effective in various combination regimens. Furthermore, retrospective analysis of the O'Shaughnessy *et al* (2002) study showed no reduction in efficacy when doses of capecitabine and docetaxel were reduced because of adverse events from the second cycle onwards (Professor C Twelves, personal communication).

While HFS was a major dose-limiting toxicity at the dosing level we used, it is important to note that grade 3 HFS is not a life-threatening adverse event. Most of the grade 2 or greater HFS was also accompanied by onycholysis, which is a well-known adverse effect of docetaxel that could be augmented by capecitabine. Clearly, as mentioned above, the solution to these issues is to reduce the doses of both agents in further studies of the combination. Indeed, we are currently investigating this combination regimen with a reduced dose of capecitabine (1000 mg m^−2^ twice daily). However, reducing the docetaxel dose should further improve safety and additional studies will be carried out to evaluate different dosing regimens in AGC.

In conclusion, capecitabine plus docetaxel is highly active in patients with previously untreated AGC. Further studies of this combination are clearly warranted, albeit with lower doses of both agents to reduce the rate of HFS and onycholysis. Capecitabine alone is clearly unique among currently available treatments for AGC in that it is compatible with oral, patient-oriented, home-based therapy.
